# Potential chemopreventive effects of Broccoli extract supplementation against 7, 12 dimethyl Benz(a)anthracene (DMBA) -induced toxicity in female rats

**DOI:** 10.1038/s41598-023-43629-2

**Published:** 2023-10-11

**Authors:** Aya M. Allam, Huda O. AbuBakr, Aya M. Yassin, Ahmed S. Abdel-Razek, Marwa S. Khattab, Eman M. Gouda, Said Z. Mousa

**Affiliations:** 1 Department of Biochemistry and Molecular Biology, Faculty of Veterinary Medicine, Cairo University, Giza, 12211 Egypt; 2 Department of Microbial Chemistry, Genetic Engineering and Biotechnology Research Division, National Research Centre, Dokki- Giza, Egypt; 3 Department of Pathology, Faculty of Veterinary Medicine, Cairo University, Giza, Egypt

**Keywords:** Biochemistry, Molecular biology, Health occupations

## Abstract

Dietary components have recently received rapidly expanding attention for their potential to halt or reverse the development of many oxidative stress-mediated diseases after exposure to environmental toxicants. 7, 12 dimethylbenz(a)anthracene (DMBA) is one of the most common environmental pollutants. The present study aimed to evaluate the chemo-preventive effects of broccoli as a nutritional component against DMBA intoxication in rats. A daily dose of aqueous (1 ml/rat) and methanolic (150 mg/kg) broccoli extracts, respectively, was given to 50-day-old female rats for 26 successive weeks after carcinogen intoxication with a single dose of 20 mg/ml of DMBA. DMBA intoxication resulted in a redox imbalance (a decreased GSH level and an increased MDA level) and increased DNA fragmentation in the liver, kidney, and brain. Besides, it affected the level of expression of the bcl2 gene in the liver, kidney, and brain tissue but didn’t affect cfos gene expression accompanied by histopathological changes. The aqueous and methanolic broccoli extract supplements ameliorated the adverse effects by increasing the level of GSH, decreasing the MDA level, and reducing DNA fragmentation. Besides, broccoli extracts decreased the expression of bcl2 in the liver and brain and up-regulated bcl2 expression in the kidney, accompanied by lowering NF-κβ 65 expression in the liver and brain and γ-catenin expression in the liver and kidney. In conclusion, broccoli as a dietary component had a strong chemoprotective effect against oxidative stress, DNA damage, and genotoxicity induced by DMBA intoxication in rats.

## Introduction

Environmental pollution has existed for centuries but only started to become significant following the industrial revolution in the nineteenth century^1^. One of the most common environmental toxicants is polyaromatic hydrocarbons (PAHs). PAHs are a group of compounds consisting of two or more fused aromatic rings. Mostly formed during the incomplete incineration of organic materials such as wood, fossil fuels, petroleum products, and coal^2^. One of the most commonly found PAHs in our environment is 7,12-dimethylbenz (a) anthracene (DMBA), which is emitted during organic matter incineration as diesel exhaust and found in medicines, dyes, plastics, pesticides, wood preservation, aerosols, tobacco smoke, barbecued meat, and overheated cooking oil^3^.

DMBA has an adverse mechanism for health hazards as it produces reactive oxygen species (ROS). These ROS are the most dangerous byproducts of cellular metabolism and the main reason for many oxidative stress-mediated diseases (i.e., cancer, diabetes, metabolic disorders, atherosclerosis, and cardiovascular diseases)^4^. The imbalance between the production of ROS at a high level and the reduction in antioxidant defence mechanisms is the main cause of oxidative stress^5^. Subsequently, the level of lipid peroxidation (LPO) increases, which can be indicated by higher levels of malondialdehyde (MDA), which serves as a reliable biomarker of LPO. Superoxide dismutase (SOD) and catalase (CAT) are antioxidant enzymes that play an essential role in combating oxidative stress and protecting cells from oxidative damage^6–8^. Accidental intake of DMBA promotes the detoxifying system through the activation of the Phase I xenobiotic detoxifying mechanism, cytochrome p450^9^. Consequently, DMBA oxidised into DMBA-3–4-epoxide that covalently binds to DNA, resulting in the formation of a DMBA-DNA adduct and fragmentation, which react with both lipids and proteins, resulting in their oxidation^9^. Besides that, these phase I modified epoxides will be conjugated to endogenous glutathione (GSH) by glutathione s-transferase (GSTs), one of the Phase II xenobiotic defence mechanisms^10,11^. Subsequently, GST inhibits the detrimental effect of oxidative stress by protecting cellular macromolecules^12^. Several studies reported that DMBA mediated an apoptotic-like mechanism^13–15^.

One of the protein families that control intrinsic and extrinsic pathways is B-cell lymphoma 2 (BCL2), which controls the release of cytochrome c from damaged mitochondria^16,17^.

cFos is one of the proto-oncogenes that promote different cellular processes, including proliferation, differentiation, transformation, and apoptosis^18,19^. The deregulation of c-Fos causes numerous pathological conditions like skeletal, immunological, and neurological defects, tumour progression, and oncogenic transformation^20^.

Nutraceutical is a food that, besides its normal nutritional value, provides health benefits, including the prevention of disease or promotion of health^21^. Nutraceuticals contain phytochemicals that are responsible for the colour, taste, and aroma of foods. They protect health from environmental carcinogens by enhancing antioxidant systems and DNA-repairing enzymes and have direct effects on the fundamental hallmarks of cancer progression and metastasis^22^. Broccoli is one of the nutraceuticals that belongs to the family Brassicaceae and is rich in alkaloids, phenolic flavonoids, tannins, polyphenols, essential oils, and polypeptides. Broccoli helps in health improvement as it contains antioxidants and anti-carcinogenic compounds: polyphenols, flavonoids, glucosinolates, sulforaphane, vitamins, minerals, and selenium^23^.

Therefore, the present study aimed to evaluate the potential chemopreventive effects of aqueous and methanolic extracts of broccoli against DMBA-induced oxidation stress in rats.

## Materials and methods

All methods and experiments were carried out following relevant guidelines and regulations.

### Chemicals

7,12 dimethylbenz [a] anthracene (DMBA) was purchased from Sigma Aldrich, St. Louis, USA (Cat No. D3254), and diluted in commercial corn oil purchased from the local markets^24^. All other chemicals used were of analytical grade.

### Nutraceuticals preparation

Aqueous and methanolic broccoli extracts were prepared according to methods described by Mandelova and Totusek^25^ and Lin et al.^26^, respectively.

#### Determination of the antioxidant activity of broccoli extracts

According to Blois^27^ and Desmarchelier et al.^28^, the 1,1-diphenyl-2-picrylhydrazyl radical-scavenging activity (DPPH) was employed to test both extracts' capacity to scavenge free radicals at various concentrations (0.3%, 0.5%, and 1%). The absorbance at 517 nm was measured using a spectrophotometer (UV/LV Spectrophotometer, Jenway, England) against a blank. The percentage scavenging effect was calculated as:

$$\% \,{\text{DPPH}}\,{\text{radical}}\,{\text{scavenging}}\,{\text{activity}}\, = \,\left( {\left( {{\text{A}}0 \, {-}{\text{ A1}}} \right)/{\text{A}}0} \right)\, \times \,{1}00,$$

where; A0: the absorbance of the control (without sample), Al: the absorbance in the presence of the sample.

#### Determination of total phenolic compounds of broccoli extracts

The separation was achieved using a ternary linear elution gradient as described by Wolfe et al.^29^ with (A) HPLC-grade water 0.2% H3P04 (v/v), (B) methanol, and (C) acetonitrile by using the Agilent 1260 infinity HPLC Series (Agilent; USA), equipped with a quaternary pump. The column used is aKinetex^®^5 [Jm EVO C18 100 mm × 4.6 mm] (Phenomenex, USA). The samples and standard were injected into a volume of 20 µL and detected through VWD.

#### Antimicrobial activity assay of broccoli extracts

Antimicrobial activity testing of the crude extracts (methanolic and aqueous) of broccoli was carried out against a set of microorganisms using the agar diffusion technique. The paper-disk diffusion assay was performed as described by Abdel-Razek et al.^30^ with some modifications. Twenty mL of medium seeded with the test organism was poured into 12 cm sterile Petri dishes. After solidification, the paper disks (6 mm in diameter) were placed on inoculated agar plates and allowed to diffuse the loaded substances into the refrigerator at 4 °C for 2 h. The plates were incubated for 24 h at 35 °C. Both bacteria and yeasts were grown on a Mueller–Hinton agar: 19.5 g L^−1^ peptone, 1.5 g L^−1^ starch-soluble, and 17 g L^−1^ agar. The pH was adjusted to 7.2. After incubation, the diameters of inhibition zones were measured with a wide panel of test microorganisms comprising Gram-positive methicillin-resistant Staphylococcus aureus (MRSA), multi-drug-resistant (MDR) Gram-negative bacteria (E. coli, Pseudomonas aeruginosa, and Klebsiella pneumonia), and yeasts (C. albicans ATCC 10231, S. cerevisiae ATCC 9080). The clear zone (inhibition zone) has been measured according to EUCAST^31^). These strains are deposited in the Microbial Chemistry Department, National Research Centre (NRC), Egypt.

### Animals

Ninety young virgins Wistar female rats aged 47–50 days, weighing between 100 and 120 g, were purchased from the animal house at the National Research Centre (NRC), DOKKI, GIZA, Egypt, and supplied with a standard diet purchased from Ibex Company, Egypt, and water ad libitum*.* They were housed for 2 weeks to be acclimatised before starting the experimental study under standardised conditions (12 h light/dark period, temperature 23 ± 2 °C, and humidity 50%). The experiment was performed in strict accordance with the recommendations in the ARRIVE guidelines, followed the guide for Care and Use of Laboratory animals, and was approved by the Institutional Animal Care and Use Committee (IACUC) (Approval number: (vet Cu 28/04/2021/299), Faculty of Veterinary Medicine, Cairo University, Egypt).

### Experimental design

By the end of the acclimatisation period, animals were randomly allocated into six groups (15 rats each). Animals in all groups were treated for 26 consecutive weeks as follows: The first group served as a control and received 1 mL of corn oil once daily by gavage for 26 weeks. The second group (DMBA group) was given a single dose of diluted DMBA (20 mg/mL/rat) in corn oil by gavage with 1 ml of distilled water daily^32,33^. The 3rd group (AQ group) received aqueous broccoli juice (1 mL/rat) I/G by gavage once daily for 26 weeks^34^. The 4th group (AQ-DMBA group) received an aqueous broccoli extract 2 weeks before the single dose of DMBA and continued until the 26th week. The 5th group (Meth group) received methanolically extracted broccoli juice (1 mL/rat) by gavage (150 mg/kg) once daily for 26 weeks (El-Baz et al.^35^). The 6th group received methanolic broccoli extract (1 mL/rat) by gavage (150 mg/kg) 2 weeks before the single dose of DMBA and continued until the 26th week (Meth_DMBA group).

At the end of the experimental period (26 weeks) and under diethyl ether anesthesia, animals from all groups were euthanized by cervical dislocation, then the liver, kidney, and brain were instantly resected and washed in cold saline. Organs were divided into two parts; the first parts were stored at − 80 °C to be used for oxidant and antioxidant biomarkers, DNA fragmentation, and genes expression. While the other parts were fixed in a 10% neutral buffered formalin solution for histopathological examination.

### Oxidant/antioxidant biomarkers

#### Assessment of reduced glutathione (GSH)

The reduced glutathione (GSH) concentration (mM/mg protein) was determined according to Baker et al.^36^. 0.5 ml of tissue homogenate, 0.25 ml of 5, 5’-Dithio-bis-2-nitrobenzoic acid (DTNB), and 1.5 ml of phosphate buffer were mixed in a tube. The reduction of 5,50-dithiobis 2-nitrobenzoic acid with glutathione produced a yellow color and absorbance was measured at 412 nm by using a UNICO-UV-2100 spectrophotometer (UNICO (Shanghai) Instrument Co., Ltd., China).

The concentration of GSH in each sample was calculated by:

$${\text{GSH}}\,{\text{concentration}}\,\left( {{\text{mM}}/{\text{mg protein}}} \right)\, = \, \frac{A \times VT}{{\varepsilon \times d \times VS}}\, \times \,\left( {{1}/{\text{mg protein}}} \right),$$

where: A = Absorbance, VT = Total volume of the assay (ml), VS = Volume of the sample, ε = Extinction coefficient of GSH at 412 nm (1.36 × 10^5^ µM^−1^ cm^−1^, d = Length of light path.

#### Assessment of lipid peroxidation

Malondialdehyde concentration (MDA) (mM/mg protein) was determined according to Albro et al.^37^. 0.5 ml of tissue homogenate was mixed with 2.5 ml of 10% TCA in a centrifuged tube and placed in a boiling water bath for 20 min. Cooling in tap water, and 1 ml of distilled water was added and mixed well. Then the tubes were centrifuged at 4000 rpm for 10 min. 2 ml of the supernatant was mixed with 1 ml of 0.67% TBA, and the tubes were put in a boiling water bath for 20 min. The tubes were cooled in tap water, and the optical density was measured spectrophotometrically (UNICO-UV-2100 spectrophotometer) (UNICO (Shanghai) Instrument Co., Ltd., China) at 532 nm against a TBA blank.

MDA concentration was calculated by:

$${\text{MDA}}\,{\text{concentration}}\,\left( {{\text{mM/mg}}\,{\text{protein}}} \right)\, = \, \frac{A \times VT}{{\varepsilon \times d \times VS}}\, \times \,\left( {{1}/{\text{mg}}\,{\text{protein}}} \right),$$

where: A = Absorbance, VT = Total volume of assay (ml), VS = Volume of sample ε = Extinction coefficient of MDA at 532 nm (1.56 × 10^5^ µM^−1^ cm^−1^), d = Length of light path.

### Assessment of DNA fragmentation

DNA fragmentation was determined according to the method described by Perandones et al.^38^. Briefly, 10–20 mg were ground in 400 μL hypotonic lysis buffer and centrifuged at 3000 × *g* for 15 min at 4 °C. The supernatant was divided into 2 parts: one was used for the gel electrophoresis, and the other was used with the pellet for quantification of the percentage of fragmented DNA by the diphenylamine at 578 nm by using a UNICO-UV-2100 spectrophotometer (UNICO (Shanghai) Instrument Co., Ltd., China).

DNA fragmentation percentage in each sample was expressed by the formula:

$$\% {\text{DNA}}\,{\text{fragmentation}}\, = \,\left( {{\text{O}}.{\text{D}}\,{\text{Supernatant}}/{\text{O}}.{\text{D}}\,{\text{Supernatant}}\, + \,{\text{O}}.{\text{D}}\,{\text{Pellet}}} \right)\, \times \,{1}00.$$

### Quantitative real-time polymerase chain reaction of BCL2 and C-Fos genes

Total RNA from 100 mg of liver, kidney, and brain tissue samples was extracted using the QIAmp RNA mini kit (QIAGEN, Hilden, Germany) as indicated by the manufacturer. Total RNA purity and concentration were obtained using a nanodrop ND-1000 spectrophotometer.

The isolated RNA was used for cDNA synthesis using reverse transcriptase **(**RevertAid RT (200 U/μL)) (Thermo Scientific, Cat.No.EP0441, Waltham, USA)**.** Real-time PCR (qPCR) was performed in a total volume of 20 μL using a mixture of 1 μL cDNA, 0.5 mM of each primer (Table 1), iQ SYBR Green Premix (Bio-Rad 170–880, USA). PCR amplification and analysis were achieved using the Bio-Rad iCycler thermal cycler and the MyiQ real-time PCR detection system. Each assay includes triplicate samples for each tested cDNA and a no-template negative control; the expression relative to the control is calculated using the equation 2^−ΔΔCT^^39^.

**Table 1:** Primer sequences of reference, GST, BCL2, and c-Fos genes of Rattus norvegicus.

Target genes	Accession no	Sequence (5' to 3')	Product size
Beta-actin (reference gene)	NM_031144.3	F: 5'- AGGCTGTGTTGTCCCTGTATG -3'	275 bp
		R: 5'- GGCCATCTCTTGCTCGAAGT -3'	
BCL2	_NM_016993.1	F: 5'- GATTTCTCCTGGCTGTCTCTGAA -3'	250 bp
		R: 5' -GTGTGTGTGTGTGTGTGTGTG- 3'	
C-Fos	NM_022197.2	F: 5'-GGAGAATCCGAAGGGAAAGGAATAA -3'	184 bp
		R: 5'- CGGTGGGCTGCCAAAATAAACT -3'	

### Histopathology

Tissue specimens from the liver, kidney, and brain were fixed in 10% neutral buffered formalin, followed by dehydration, clearance, and embedding in paraffin. Tissue sections 4 m thick were made using a rotatory microtome (Leica 2135, Germany) stained with hematoxylin and eosin (H&E)^40^. Tissue sections were examined using an Olympus BX43 light microscope and photographed using an Olympus DP27 camera. Degeneration, necrosis, and leukocytic infiltration were scored on a scale from 0 to 3 in the liver and kidney. Neuronal degeneration and gliosis in the brain cortex were scored on a scale from 0 to 3, in which 0 = no detected change, 1 = mild change (0–25%), 2 = moderate change (25–50%), and 3 = severe change (50–100%). Scores were analysed by the Kruskal–Wallis test and presented in a box plot.

### Immunohistochemistry nuclear factor κβ P65 (NF- κβ) and gamma catenin

Immunohistochemistry of nuclear factor κβ P65 (NF- κβ) and gamma catenin was performed in Paraffin-embedded tissue sections. Tissue sections were deparaffinized by xylene and rehydrated by descending concentrations of ethanol. The antigen retrieval was carried out using citrate buffer (pH 6) overnight in the incubator at 70 Co for nuclear factor κβ P65 and Tris/EDTA buffer (pH 9) for half an hour for gamma catenin immunohistochemistry. Primary antibodies against NF- κβ P65 (sc-8008, Santa Cruz, USA) and gamma catenin (ab218437, Abcam, UK) were applied to slides and incubated overnight. Hydrogen peroxide solution was applied to slides to remove endogenous peroxidase, followed by the secondary HRP-labelled antibody according to manufacturer protocol (Universal poly HRP DAB kit for mouse and rabbit, Genemed, Sakura, USA). Freshly prepared DAB was applied to slides for 20 min to develop the brown colour. Washing with phosphate buffered saline (PH 7.4) twice for 5 min was applied to slides between each step^41^. Negative control slides weren’t stained with primary antibodies and were stained with secondary antibodies only^42^. The area percent of nuclear factor κβ P65 and gamma catenin were analysed by Image J software in 3 organ images per rat at 200X magnification.

### Statistical analysis

The values in medians and data visualization were obtained with R Studio statistical analysis. Data from multiple hypothesis samples were checked for their normal distribution by the "Shapiro t-test". Comparisons between different groups were carried out by analysis of variance (one-way ANOVA) by using the "F-Test" P ≤ 0.05. Error correction occurred by False discovery rate following "Benjamini-Hochberg " procedures to control the percentage of incorrect discoveries (false positives) among all rejected hypotheses. (R Studio statistical analysis software) RStudio Team^43^.

### Ethics approval and consent to participate

The study was performed in strict accordance with the recommendations in the ARRIVE guidelines and followed the guide for Care and Use of Laboratory Animals and was approved by the Institutional Animal Care and Use Committee (IACUC) (Approval number: (vet Cu 28/04/2021/299), Faculty of Veterinary Medicine, Cairo University, Egypt.

## Results

### Antioxidant activity of the aqueous and methanolic broccoli extract findings

The antioxidant activity of broccoli was evaluated using the DPPH free radical scavenging test. The DPPH radical scavenging activities of methanolic and aqueous extracts of broccoli at different concentrations are presented in Tables 3 and 4. The result of this study revealed that methanolic and aqueous extracts possessed up to 81.60% and 71.53% of DPPH radical scavenging activity at a concentration of 1% and 100% of dried extracts, respectively. The methanol extract of broccoli was found to possess a much higher radical scavenging activity than the water extract. In the present study for the evaluation of DPPH radical scavenging activity, the concentration was expressed by the ratio of crude sample per solvent volume. Based on these concentrations, our municipal broccoli is estimated to have higher DPPH radical scavenging activity (Table 2).

**Table 2:** % DPPH Radical-Scavenging activity of aqueous and methanolic broccoli extract.

% DPPH radical-scavenging activity of methanolic broccoli extract			
Dried extract concentration %	0.3%	0.5%	1%
DPPH radical-scavenging activity %	35.42	72.92	81.60
% DPPH radical-scavenging activity of aqueous broccoli extract			
Dried extract concentration %	10%	50%	100%
DPPH radical-scavenging activity %	29.86	57.64	71.53

### The phytochemical components of aqueous and methanolic broccoli extracts findings

**Figure 1:**
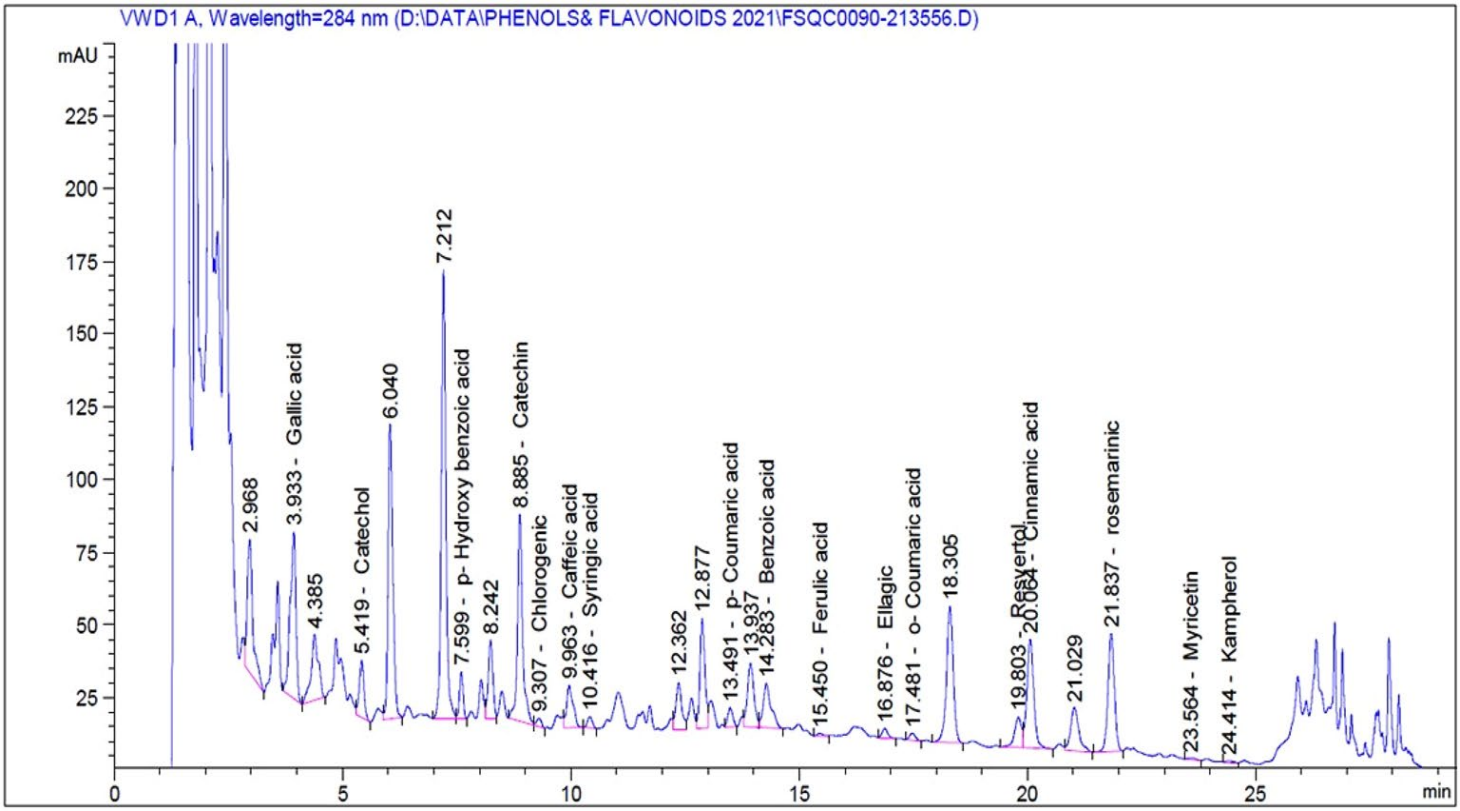
The spectrum of identified compounds from aqueous broccoli juice by using LCMS.

**Figure 2:**
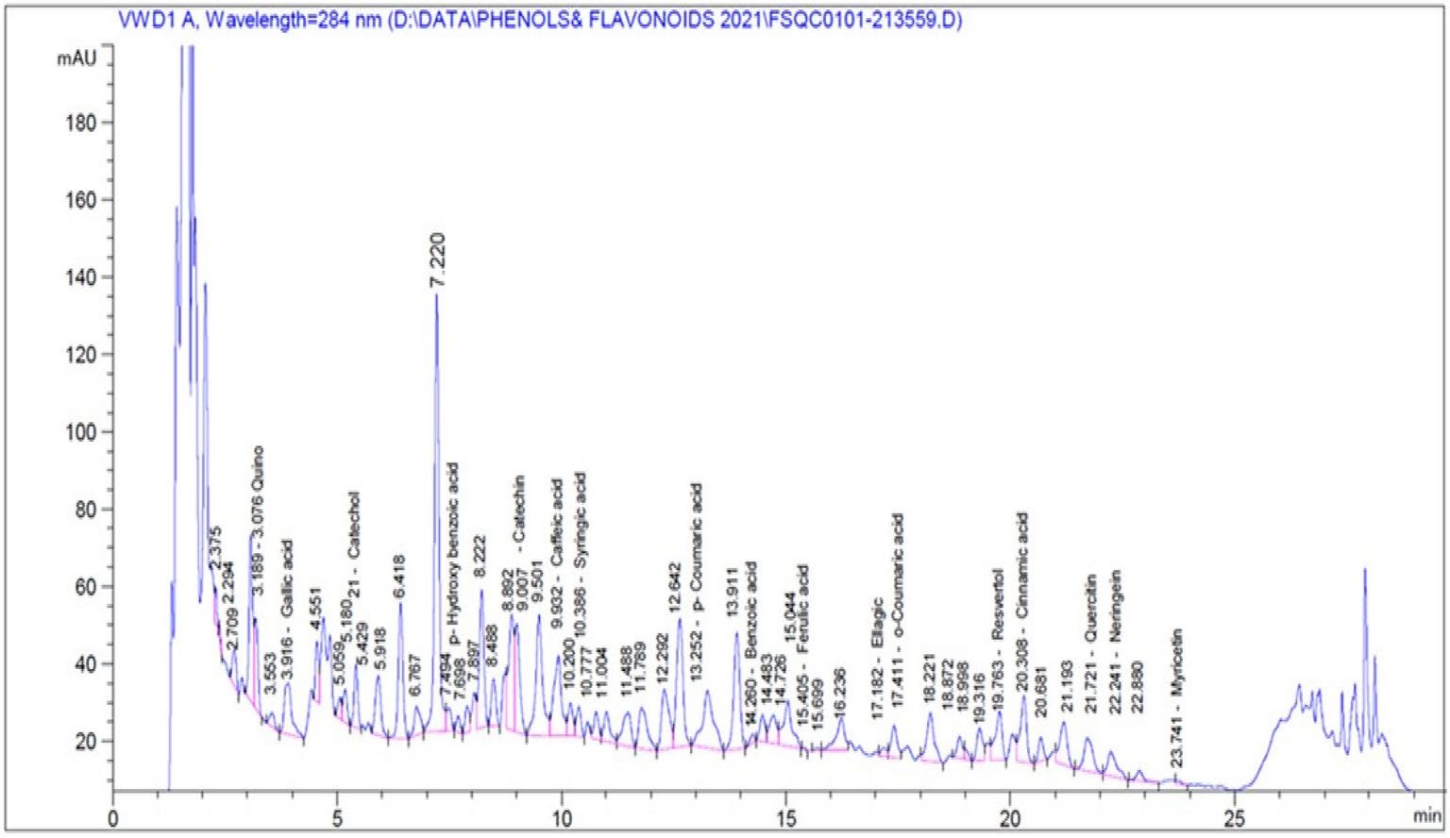
The spectrum of identified compounds from methanolic broccoli extract by using LCMS.

Phytochemical analysis was performed using 1 cms Liquid chromatography-mass spectrometry indicating the presence of pyrogallol, 3-hydroxytyrosol, vanillic acid, and rutin in trace amounts in both aqueous and methanolic extracts. In the methanolic extract, quinol, gallic acid, catechol, p-hydroxybenzoic acid, catechin, caffeic acid, syringic acid, p-coumaric acid, benzoic acid, o-coumaric acid, resveratrol, cinnamic acid, quercitin, naringenin, and myricetin were present in larger amounts than the aqueous extracts (Figs. 1, 2 and Table 3).

**Table 3:** Identified compounds from broccoli extracts.

Aqueous extract			Methanolic extract		
RT (min)	Amount (mg/Kg)	Name	RT (min)	Amount (mg/Kg)	Name
2.900	–	Pyrogallol	2.900	–	Pyrogallol
3.170	–	Quinol	3.189	92.35009	Quinol
3.933	2.98251	Gallic acid	3.916	32.58962	Gallic acid
4.760	–	3-Hydroxytyrosol	4.760	–	3-Hydroxytyrosol
5.419	3.09817	Catechol	5.412	72.95418	Catechol
7.599	1.88783	P-Hydroxy Benzoic acid	7.698	17.26283	P-Hydroxy Benzoic acid
8.885	1.83677	Catechin	8.892	28.87423	Catechin
9.307	1.51242e − 1	Chlorogenic acid	9.300	–	Chlorogenic acid
9.800	–	Vanillic acid	9.800	–	Vanillic acid
9.963	5.78420e − 1	Caffeic acid	9.932	34.34111	Caffeic acid
10.416	1.70520e − 1	Syringic acid	10.386	11.28222	Syringic acid
13.491	1.56673e − 1	P-Coumaric acid	13.252	34.68773	P-Coumaric acid
14.283	19.15777	Benzoic acid	14.260	51.29684	Benzoic acid
15.450	4.64464e − 2	Ferulic acid	15.405	4.31409e-1	Ferulic acid
16.700	–	Rutin	16.700	–	Rutin
16.876	2.46078e − 1	Ellagic acid	17.182	5.46585	Ellagic acid
17.481	1.16326e − 1	O-Coumaric acid	17.411	9.54896	O-Coumaric acid
19.803	5.92041	Resvertol	19.763	250.41168	Resvertol
20.064	1.31446	Cinnamic acis	20.308	17.72024	Cinnamic acid
21.600	–	Quercitin	21.721	263.07135	Quercitin
21.837	39.45981	Rosemarinic	22.000	–	Rosemarinic
22.400	–	Neringein	22.241	272.08179	Neringein
23.564	9.31744e − 1	Myricetin	23.741	34.76430	Myricetin
24.414	3.72962e − 1	Kaempferol	24.700	–	Kaempferol

*RT* Retention time, (–) means trace amount.

### Antimicrobial activity assay of broccoli extracts

In the antimicrobial activity assay for crude extracts obtained from broccoli, we have used a set of microorganisms, including gram-positive and gram-negative bacteria and yeasts. Among these tested strains were two multi-drug-resistant gram-negative *P. aeruginosa* and *K. pneumonia* and Gram-positive methicillin-resistant *Staphylococcus aureus* (MRSA). Based on the disk-diffusion agar method, the aqueous extract showed no activity against all tested pathogens. In contrast, the methanolic extract exhibited potent activities compared with different standards. Gentamicin (CN) was used as a standard for both Gram-positive and Gram-negative bacteria, with colistin sulphate (CT) as a standard for MDR Gram-negative *P. aeruginosa* and *K. pneumoniae* and vancomycin as a standard for the MRSA strain. On yeast plates, we used amphotericin B (AMB). The methanolic extract showed high activity against the tested bacteria *P. aeruginosa* (17.5 mm), and *K. pneumonia* (24 mm), and MRSA (23 mm) compared to their standards. In addition to its potent antibacterial activity, the methanolic extract of our domestic broccoli showed very high antifungal activity against the tested *Candida albicans* (15 mm) compared to Amphotericin B, which showed no activity against the same strain but showed fungistatic activity against Saccharomyces cerevisiae (Table 4).

**Table 4:** Antimicrobial activities of the methanolic and aqueous extracts of broccoli in agar diffusion assay.

	*E. coli*	Pseudomonas aeruginosa*	Klebsiella pneumonia*	Staphylococcus aureus**	*Candida albicans*	*Saccharomyces cerevisiae*
Colistin sulphate (CT)	12	14	12	NA	NA	NA
Vancomycin (VA)	NA	NA	NA	29	NA	NA
Gentamicin (CN)	–	–	–	–	NA	NA
Amphotericin B (AMB)	NA	NA	NA	NA	–	–
Methanolic extract	–	17.5	24	23.5	15	±
Aqueous extract	–	–	–	–	–	–

*MDR strain; **MRSA; *NA* not applicable; ( −) No activity; ( ±) Fungistatic activity; Clear Zone (mm).

### Oxidant/antioxidant biomarkers findings

**Figure 3:**
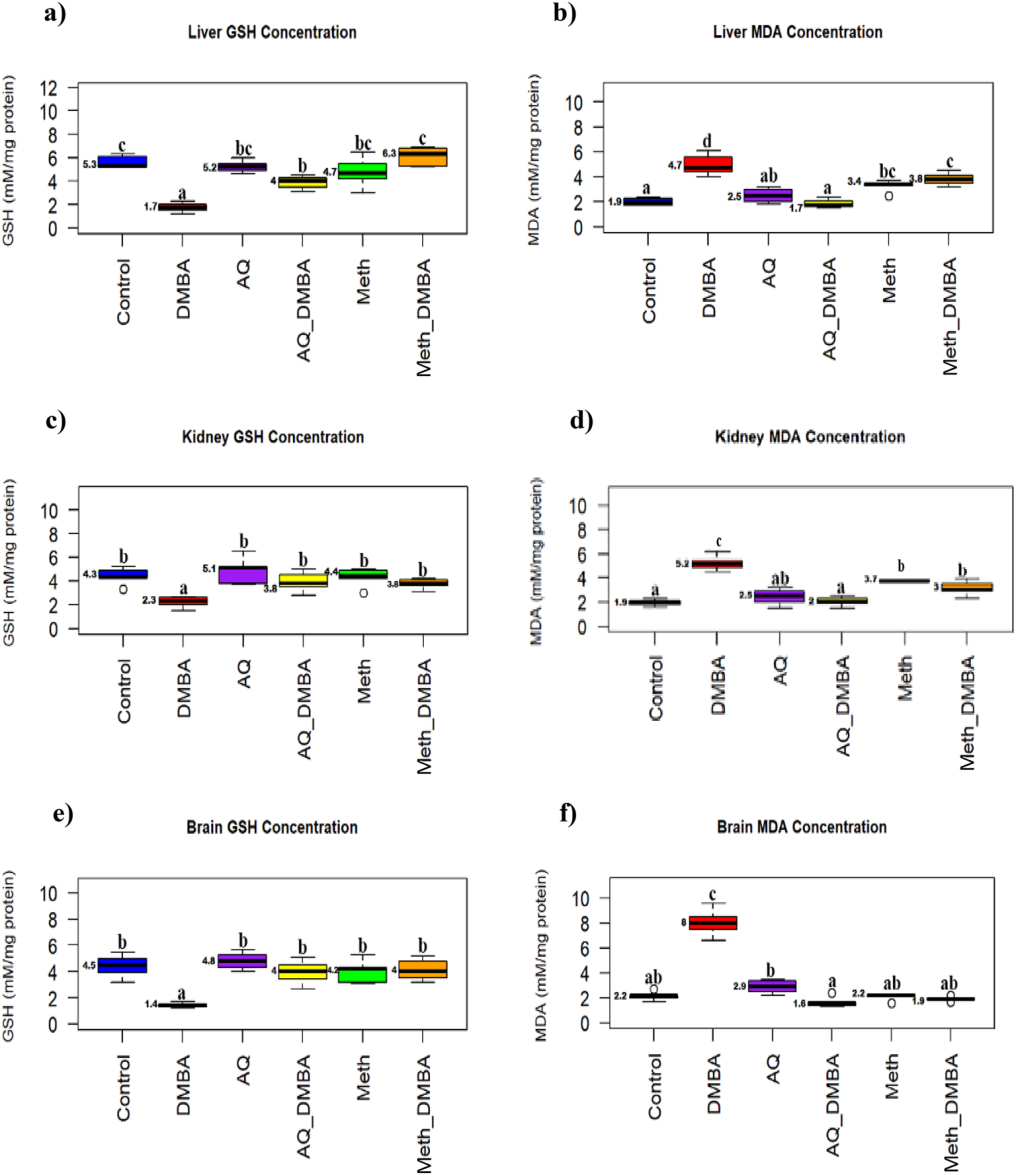
Oxidant/antioxidant biomarkers in different groups. Values are medians, n = 5. Boxplots carrying different letters (a, b, and c) are significantly different at P ≤ 0.05. (**a**) Reduced glutathione (GSH) mM/mg protein concentration in liver (**b**) Malondialdehyde (MDA) mM/mg protein concentration in liver (**c**) Reduced glutathione (GSH) mM/mg protein concentration in kidney (**d**) Malondialdehyde (MDA)mM/mg protein concentration in the kidney. (**e**) Reduced glutathione (GSH) mM/mg protein concentration in the brain (**f**) Malondialdehyde (MDA) mM/mg protein concentration in the brain.

The concentration of GSH was significantly decreased in the DMBA group in the liver, kidney, and brain in comparison to the control. Meanwhile, co-supplementation with broccoli extracts returns its concentration nearly to its normal value (Fig. 3a,c,e). While the concentration of MDA was significantly increased in the DMBA group in the liver, kidney, and brain in comparison to the control. While co-supplementation with broccoli extracts returned its concentration nearly to its normal value (Fig. 3b,d,f).

### DNA fragmentation findings

**Figure 4:**
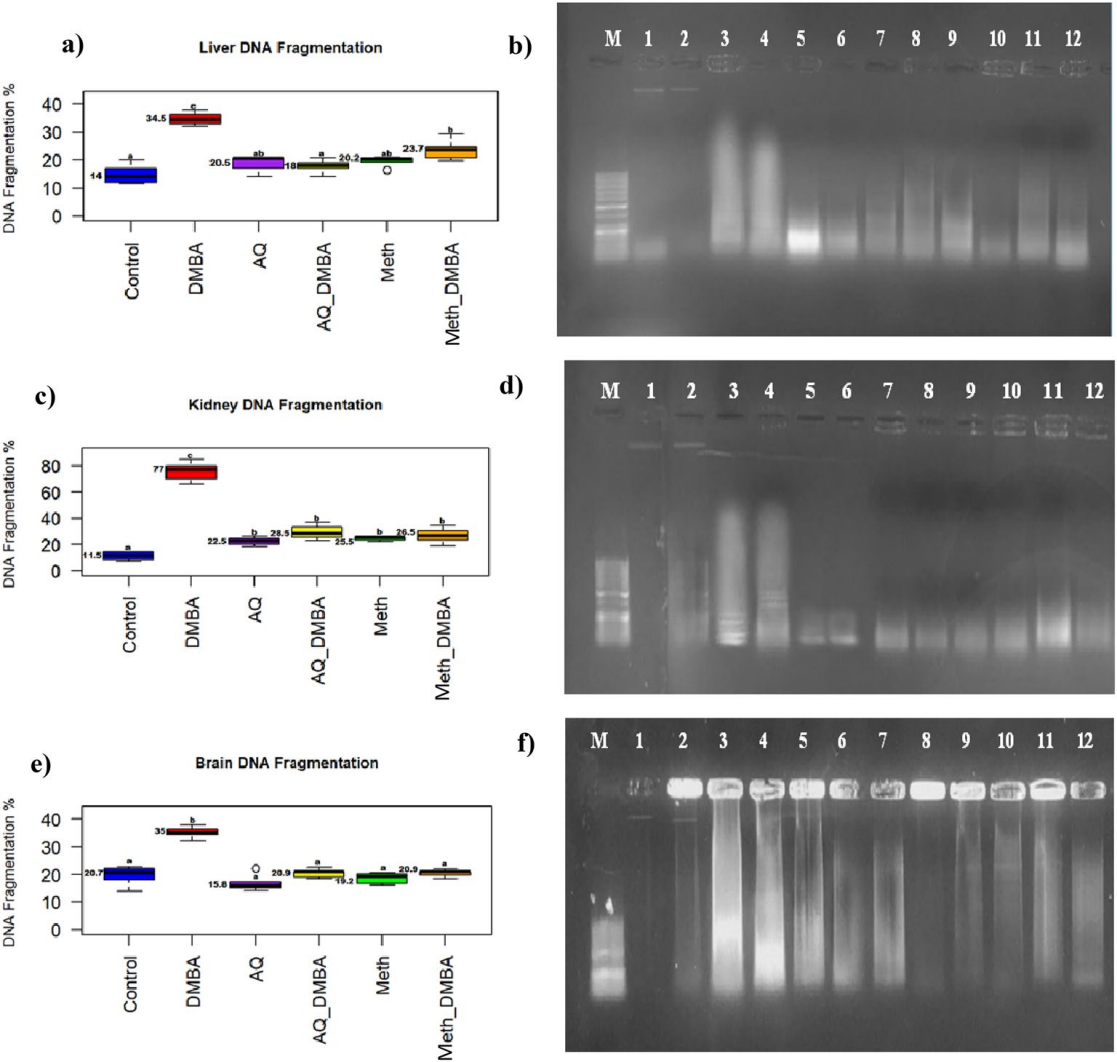
DNA fragmentation % in different groups. (**a**) Liver DNA fragmentation percentage. Values are medians, n = 5. Boxplots carrying different letters (a, b, and c) are significantly different at P ≤ 0.05 (**b**) Electrophoretic mobility of liver fragmented DNA in different groups on 1% uncropped agarose gel. (**c**) Kidney DNA fragmentation percentage. Values are medians, n = 5. Boxplots carrying different letters (a,b, and c) are significantly different at P ≤ 0.05. (**d**) Electrophoretic mobility of kidney fragmented DNA in different groups on uncropped 1% agarose gel. (**e**) Brain DNA fragmentation percentage. Values are medians, n = 5. Boxplots carrying different letters (a,b, and c) are significantly different at P ≤ 0.05. (**f**) Electrophoretic mobility of brain fragmented DNA in different groups on uncropped 1% agarose gel. lane 1,2: control; lane 3,4: DMBA; lane 5,6: AQ; lane 7,8: AQ_DMBA; lane 9,10: Meth, lane 11,12: Meth_DMBA; M: DNA ladder. Supplementary Fig. S1. Electrophoretic mobility of different organs of fragmented DNA in different groups on 1% uncropped agarose gel.

The percentage of DNA fragmentation was significantly increased in the DMBA group in the liver, kidney, and brain in comparison to the control. Herein, co-supplementation with broccoli extracts decreased this percentage nearly to the control value. Agarose gel electrophoresis of DNA revealed significant DNA fragmentation in DMBA-treated rats tissues, whereas the co-supplementation of broccoli decreased fragmentation significantly (Fig. 4). Supplementary Fig. S1 showed the electrophoretic mobility of different organs of fragmented DNA in different groups on a 1% uncropped agarose gel.

### Bcl2 gene expression findings

**Figure 5:**
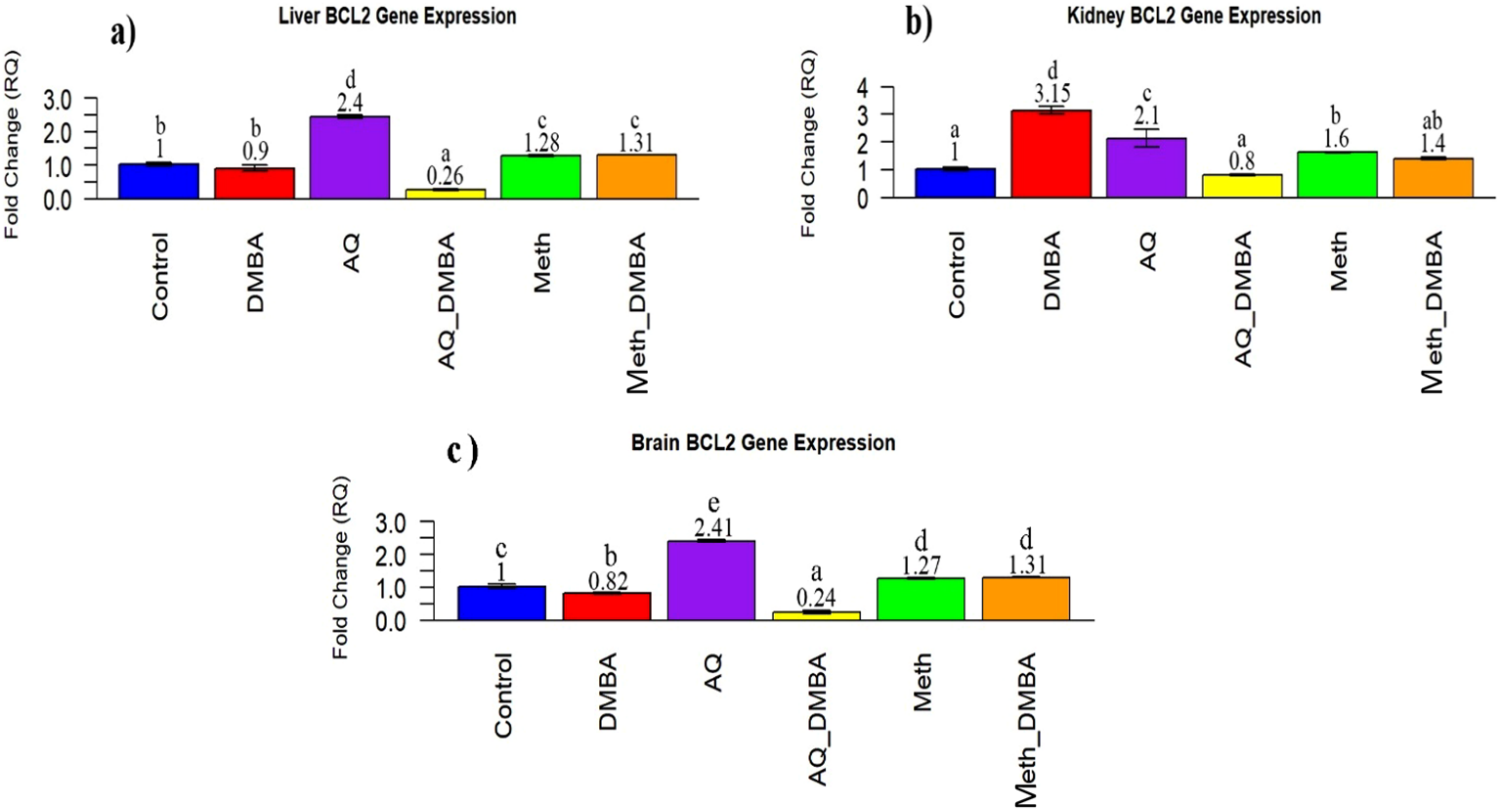
Quantitive RT-PCR of BCL2 GENE expression in different organs in various groups. (**a**). Evaluation of bcl2 gene expression in the liver in groups compared with control and other extracts group. values expressed mean $\pm$ SE n = 5. Bar carrying different letters (a,b,c,d,e) are significantly different at p $\le$ 0.05. (**b**). Evaluation of bcl2gene expression in the kidney in groups compared with control and other extracts group. values expressed mean $\pm$ SE n = 5. Bar carrying different letters (a,b,c,d,e) are significantly different at p $\le$ 0.05. (**c**) Evaluation of bcl2 gene expression in the brain in groups compared with control and other extracts group. values expressed mean $\pm$ SE n = 5. Bar carrying different letters (a,b,c,d,e) are significantly different at P ≤ 0.05.

In the liver, the expression of the Bcl2 gene showed a non-significant change in the DMBA-treated group in comparison to the control. However, its expression after co-administration with aqueous broccoli extract showed a sharp decrease in comparison to the control. While co-supplementation with methanolic extract showed a significant elevation in expression compared to control (Fig. 5a). In the kidney, the expression of the Bcl2 gene significantly increased in the DMBA-treated group in comparison to the control. While co-supplementation with both broccoli extracts caused a significantly decreased level near the control value (Fig. 5b). In the brain, the expression of the Bcl2 gene significantly decreased in the DMBA-treated group in comparison to the control. However, its expression in the AQ-DMBA group significantly decreased and in the Meth-DMBA group significantly increased in comparison to the control (Fig. 5c).

### c-Fos gene expression findings

**Figure 6:**
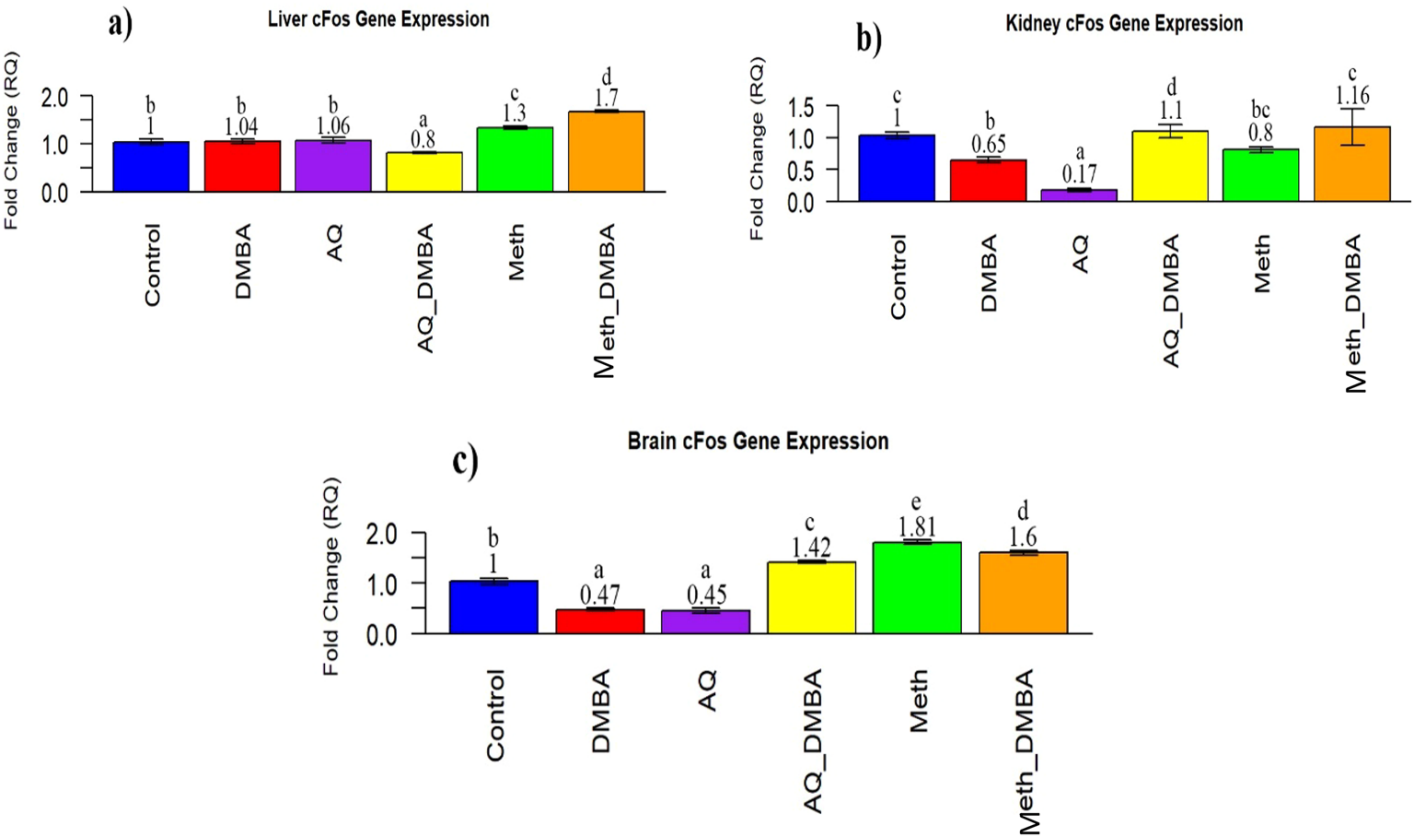
Quantitive RT-PCR of CFOS GENE expression in different organs in various groups. (**a**). Evaluation of c-FOS gene expression in the liver in groups compared with control and other extracts group. values expressed mean $\pm$ SE n = 5. Bar carrying different letters (a,b,c,d,e) are significantly different at p $\le$ 0.05. (**b**). Evaluation of c-FOSgene expression in the kidney in groups compared with control and other extracts group. values expressed mean $\pm$ SE n = 5. Bar carrying different letters (a,b,c,d,e) are significantly different at p $\le$ 0.05. (**c**) Evaluation of cFOSgene expression in the brain in groups compared with control and other extracts groups. values expressed mean $\pm$ SE n = 5. Bar carrying different letters (a,b,c,d,e) are significantly different at P ≤ 0.05.

In the liver, the expression of the c-fos gene showed a non-significant change in the DMBA- treated group in comparison to the control. However, its expression after co-administration with aqueous broccoli extract, it was significantly decreased, and in the methanolic extract was significantly increased in comparison to the control (Fig. 6a). In the kidney, the expression of the c-Fos gene significantly decreased in the DMBA-treated group in comparison to the control. While co-supplementation with both broccoli extracts significantly increased in comparison to the control (Fig. 6b). In the brain, the expression of the c-fos gene significantly decreased in the DMBA- treated group in comparison to the control. However, its expression after co-administration with methanolic and aqueous broccoli extracts significantly increased in comparison to the control (Fig. 6c).

### Histopathology findings

**Figure 7:**
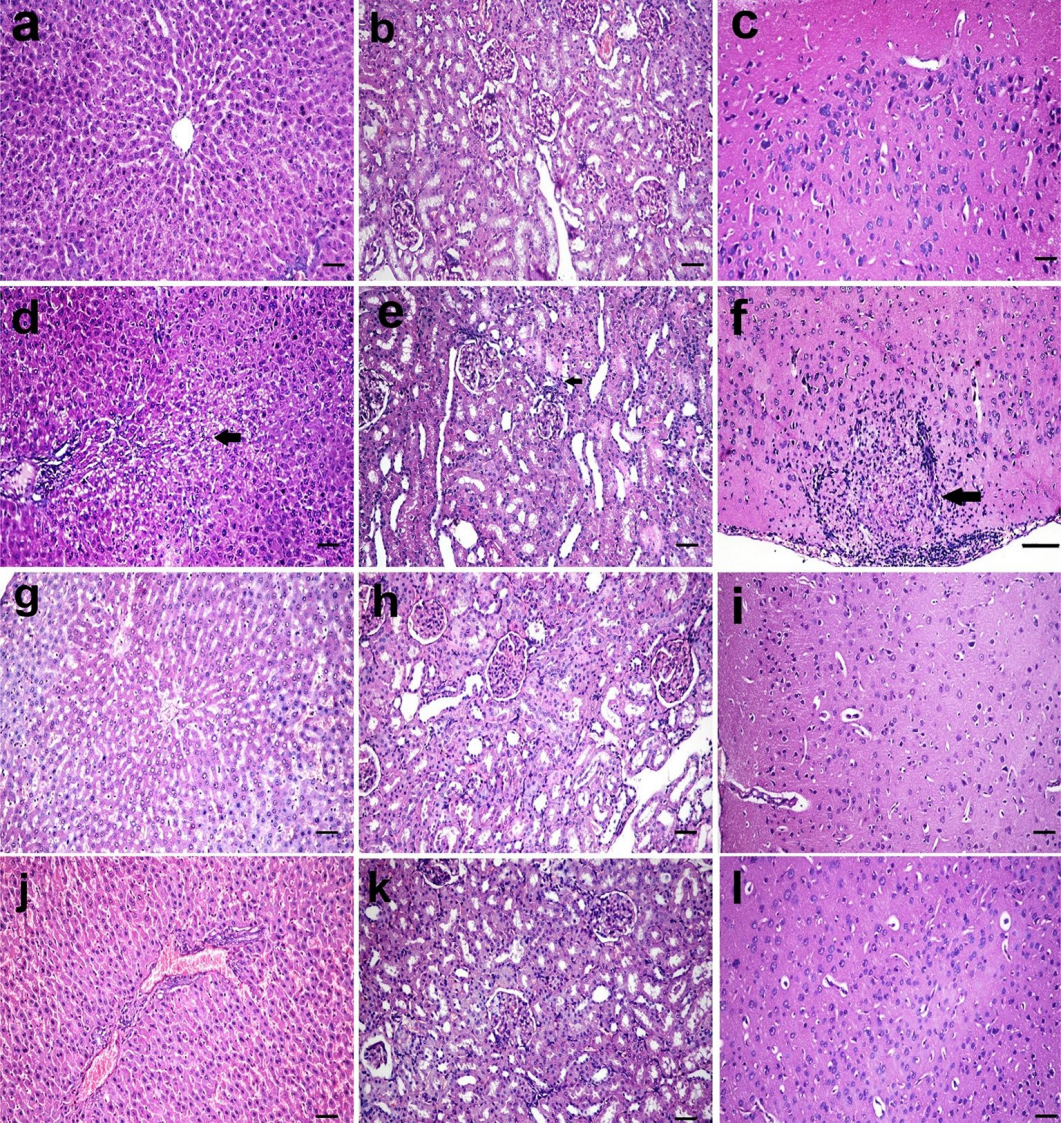
(**a**) Liver, (**b**) kidney, and (**c**) brain of rats in the control group showing normal histological structure. In DMBA treated rats, the (**d**) liver showed periportal focal vacuolation and apoptosis of hepatocytes (arrow), (**e**) the kidney showed mild degeneration in the tubular epithelium (arrow), (**f**) focal gliosis, and meningitis in the cortical hemisphere (arrow). In DMBA-aqueous broccoli-treated rats, (**g**) the liver, (**h**) kidney, and (**i**) brain showed mild histopathological alteration. In DMBA-methanolic broccoli extract treated rats, (**j**) the liver, (**k**) kidney, and (**l**) brain showed mild histopathological alteration. (scale bar = 50 µm).

**Figure 8:**
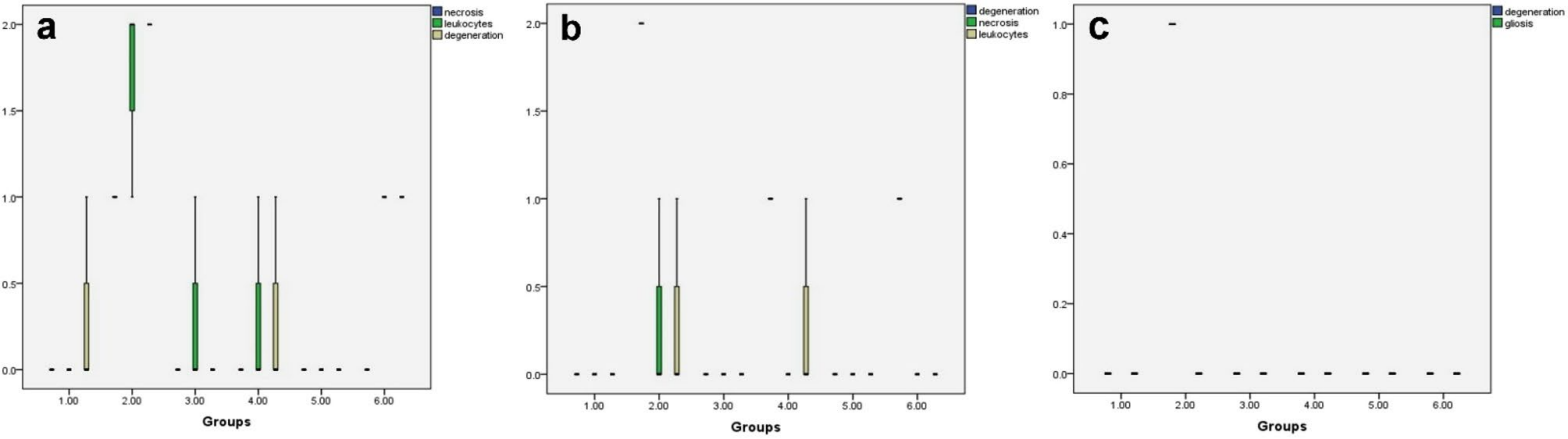
Boxplot of lesion scores in (**a**) liver, (**b**) kidney and (**c**) brain. (**a**) scores of necrosis, leukocytic infiltration and hepatocytes degeneration in liver, (**b**) scores of degeneration, necrosis and leukocytes infiltration in kidney, (**c**) scores of neuronal degeneration and gliosis in brain. The boxes are the interquartile range (IQR). The medians are the thick middle lines. The maximum and minimum values are represented by the thin horizontal lines at the top and bottom.

Microscopy of the liver, kidney, and brain in rats of the control group, aqueous broccoli group, and methanolic broccoli extract group revealed normal histological structure (Fig. 7a–c). Microscopy of the liver in DMBA treated rats showed piecemeal necrosis in which periportal hepatocytes were destroyed and lymphocytic infiltration was seen at the interface between the portal tracts and the limiting plate of periportal hepatocytes (interface hepatitis). Focal lytic necrosis, apoptosis, and focal inflammation were observed in the hepatic parenchyma. Dilatation of hepatic sinusoids, leucocytosis, and kupffer cell hyperplasia was also observed. The hepatocytes were hypertrophied with occasional binucleation and karyomegaly (Fig. 7d). The kidneys of DMBA- group rats showed slight degeneration of tubular epithelium and a few mononuclear cell infiltrations (Fig. 7e). The brain showed focal gliosis and meningitis in the cortical hemisphere and mild neuronal degeneration (Fig. 7f). These lesions observed in the liver, kidney, and brain were remarkably decreased in the DMBA-aqueous broccoli group (Fig. 7g–i) and the DMBA-methanolic broccoli extract group (Fig. 7j–l). The lesion scores of liver, kidney and brain in different groups are represented in a boxplot (Fig. 8). The hepatocyte necrosis and degeneration were significantly increased in DMBA group compared to control, whereas they were significantly decreased in DMBA-aqueous broccoli group and the DMBA-methanolic broccoli extract group. Necrosis of the renal tubular epithelium and leukocytic infiltration were significantly increased in DMBA group compared to the control. However, tubular necrosis recorded a significant decrease in DMBA-aqueous broccoli group and the DMBA-methanolic broccoli extract group compared to DMBA group. The neuronal degeneration score was significantly increased in DMBA group compared to other groups.

### Immunohistochemistry findings

**Figure 9:**
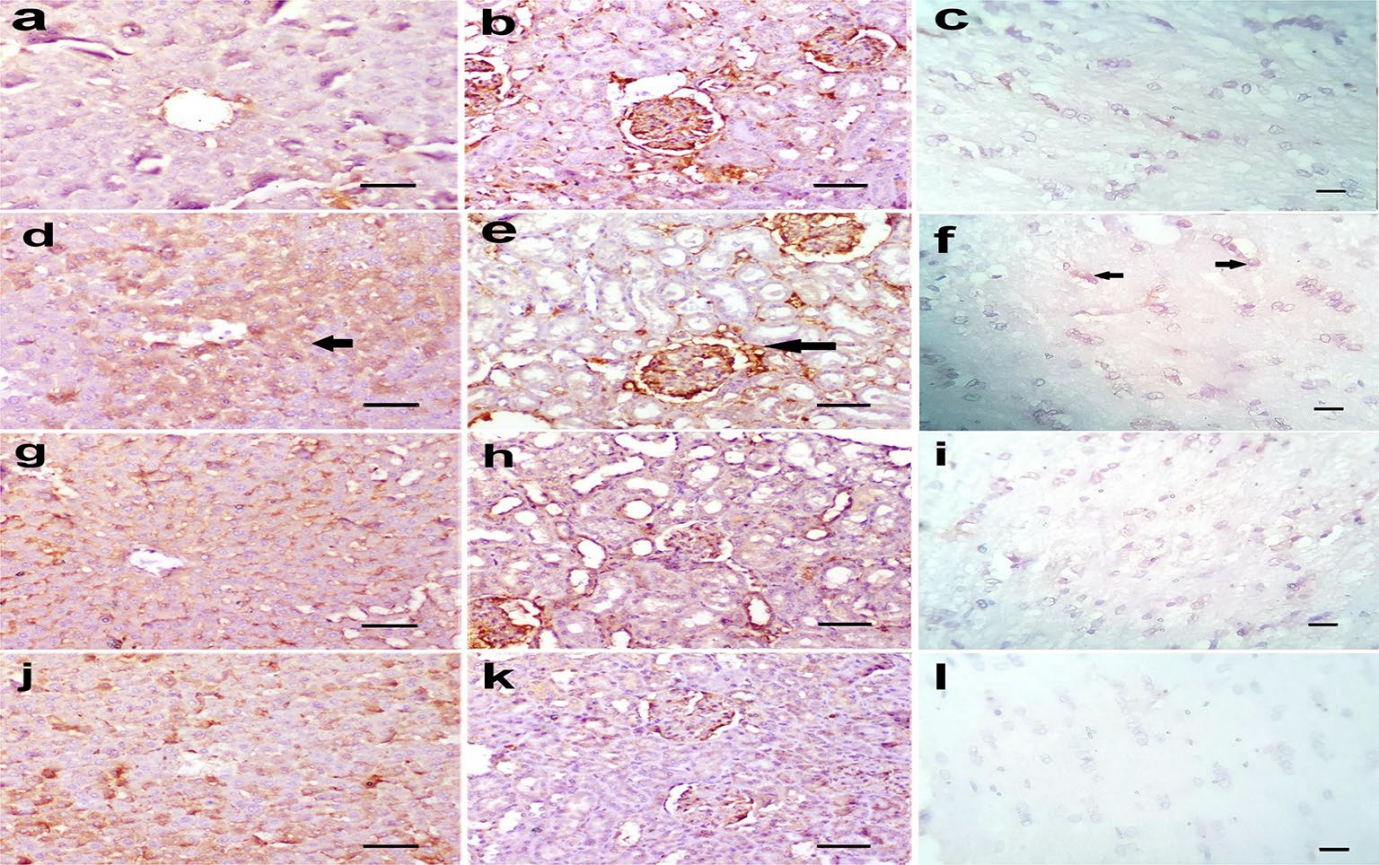
The expression of NF-κβ P65 in (**a**) liver, (**b**) kidney, and (**c**) brain of rats in the control group. In DMBA treated rats, the NF-κβ P65 was highly expressed in (**d**) hepatocytes of the liver (arrow), (**e**) glomerular cells in the kidney (arrow), (**f**) and glia cells in the brain (arrow). In DMBA-aqueous broccoli-treated rats, the NF-κβ P65 was mildly expressed in the (**g**) liver, (**h**) kidney, and (**i**) brain. In DMBA-methionine treated rats, the NF-κβ P65 was mildly expressed in (**j**) the liver, (**k**) the kidney, and (**l**) the brain. Immunoperoxidase stain (scale bar = 50 µm).

The expression of NF-κβ P65 in the liver, kidney, and brain of rats in the control group, aqueous broccoli group, and methanolic group was similar and recorded no significance. The expression of NF-κβ P65 was significantly increased in the liver and brain of rats in the DMBA group compared to the control group; the highest expression was recorded in the liver. The expression was significantly decreased in the liver, kidney, and brain of rats in the DMBA-aqueous broccoli group and the DMBA methanolic group, except in the kidney (Figs. 9 and 11a).

**Figure 10:**
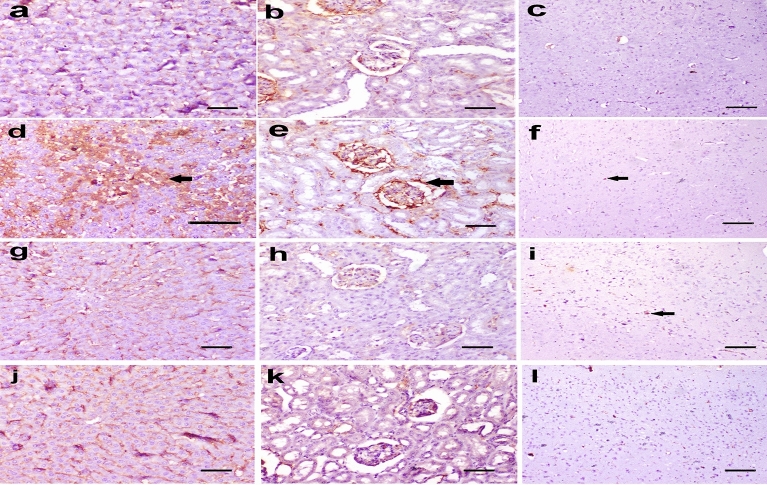
The expression of gamma catenin in (**a**) liver, (**b**) kidney, and (**c**) brain of rats in the control group. In DMBA treated rats, the gamma catenin was highly expressed in (**d**) hepatocytes of the liver (arrow), (**e**) glomerular cells in the kidney (arrow), (**f**), and not expressed in the brain. In DMBA-aqueous broccoli treated rats, the gamma catenin was mildly expressed in the (**g**) liver, (**h**) kidney, and (**i**) not expressed in the brain (arrow). In DMBA-methanolic broccoli treated rats, the gamma catenin was mildly expressed in (**j**) the liver, (**k**) the kidney, and (**l**) not expressed in the brain. Immunoperoxidase stain (scale bar = 50 µm).

**Figure 11:**
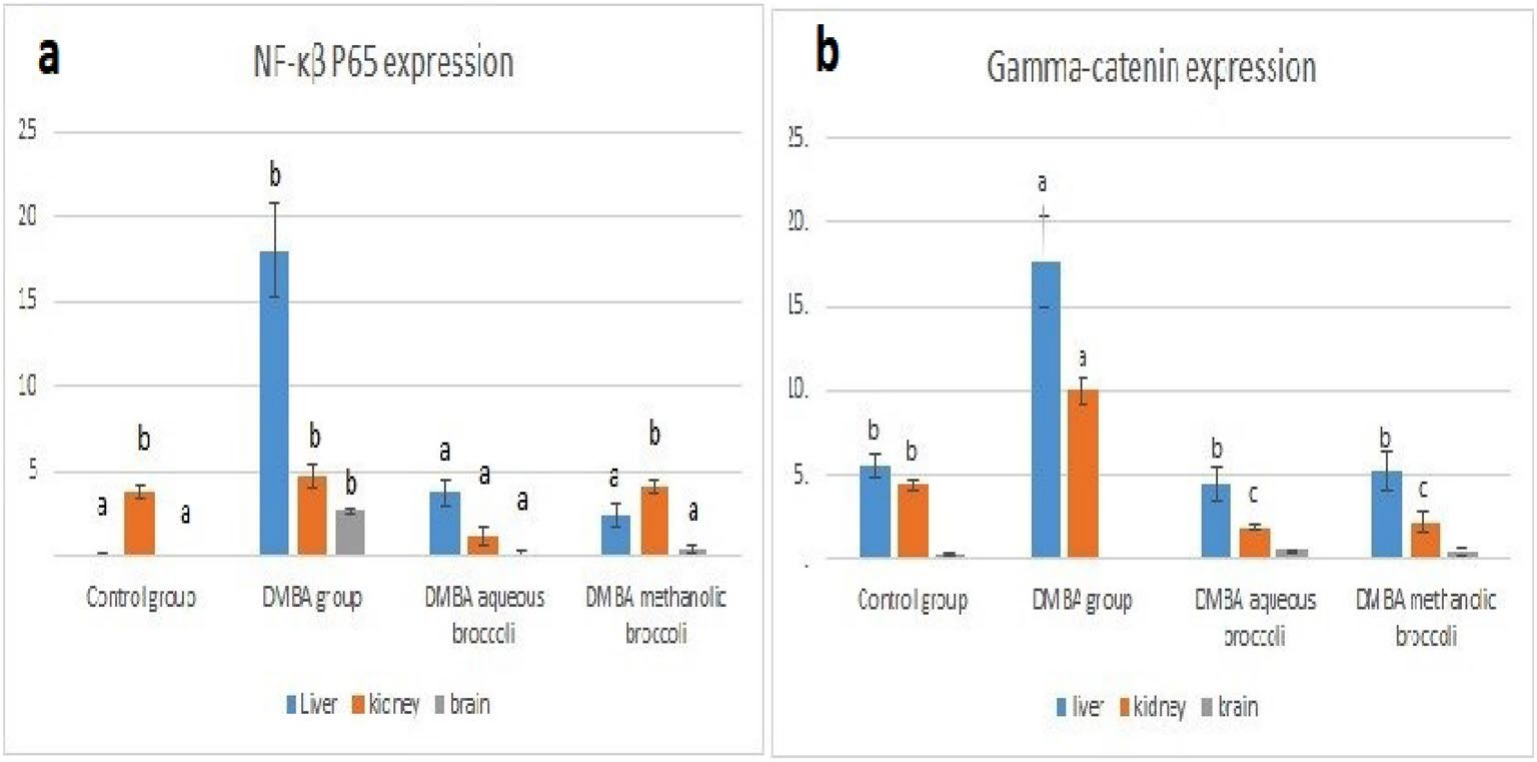
The expression of NF-κβ P65 and gamma catenin in different organs. (**a**) the expression of NF-κβ P65 in liver, kidney and, brain in DMBA, DMBA aqueous and DMBA methanolic extracts in comparison to control. (**b**) the expression of gamma catenin in liver, kidney and, brain in DMBA, DMBA aqueous and DMBA methanolic extracts in comparison to control. Bars represent the mean value ± standard error of area percent of NF-κβ P65 and gamma catenin immunohistochemistry. Bars bearing different lowercase letters are significant.

The expression of γ-catenin was significantly increased in the hepatocytes of the liver and glomerular cells of the kidney in the DMBA group compared to the control. Its expression was, however, decreased in the DMBA-aqueous broccoli group and the DMBA-methanolic broccoli group. The expression of γ-catenin in the brain recorded no significant difference between groups (Figs. 10 and 11b).

## Discussion

The unnatural synthetic drugs that are used in medication usually provoke various side effects that force different clinical and experimental studies to utilize natural products^35,44^. For instance, our target in the present study is to use a nutraceutical substance, a green chemoprotective, to ameliorate the DMBA oxidative stress mechanism. Broccoli is one of the famous nutraceutical foods used in the prevention of disease incidence through its pronounced phytochemical contents^21^. Phenolics have antioxidant capacity and may protect the cells against oxidative damage. Flavonoids are a group of polyphenolic compounds with known properties that include free radical scavenging, inhibition of hydrolytic and oxidative enzymes, and anti-inflammatory action^45^. In the present study, the analysis of phytochemicals revealed the presence of pyrogallol, 3-hydroxytyrosol, vanillic acid, rutin, quinol, gallic acid, catechol, p-hydroxybenzoic acid, catechin, caffeic acid, syringic acid, p-coumaric acid, benzoic acid, o-coumaric acid, resveratrol, cinnamic acid, quercitin, naringenin, kampherol, and myricetin.

In the methanolic extract, quinol, gallic acid, catechol, p-hydroxybenzoic acid, catechin, caffeic acid, syringic acid, p-coumaric acid, benzoic acid, o-coumaric acid, resveratrol, cinnamic acid, quercitin, naringenin, and myricetin were present in larger amounts than in the aqueous extracts. These findings were in agreement with those of Kim et al.^46^, in which the methanolic crude extract for broccoli sprouts contained abundant amounts of phenolic compounds such as rutin, quercetin, chlorogenic acid, catechin, and p-coumaric acid, followed by 4-hydroxybenzoic acid, sinapic acid, epicatechin, and caffeic acid.

Paśko et al.^47^ reported that robinin, a kampherol glucoside, was the only flavonoid identified in broccoli sprouts, while our findings confirmed the presence of kampherol and rutin as the flavonoid parts in the whole broccoli extract. Pająk et al.^48^ also stated that kaempferol is the main compound in broccoli sprouts after hydrolysis. As well as Hwang and Lim^49^, reported that the total phenolic contents of an 80% methanol extract of broccoli were greater than the aqueous extract. Several studies have suggested that ORAC (The Oxygen Radical Absorbance Capacity) could be attributed to the total phenolic content of medicinal plants^50^.

Besides that, the in vitro evaluation of the antioxidant activity of broccoli in our results revealed that it has high DPPH radical scavenging activity. These results are in line with those of Guo et al.^51^ and Kim et al.^46^, who reported that broccoli could serve as a free radical scavenger against DPPH, acting possibly as a primary antioxidant, as well as a dietary supplement to minimize oxidative stress.

Also, Bahgat et al.^52^ reported that both methanol and water could give the broccoli extracts a higher reducing power. In terms of ferrous ion chelating ability and DPPH radical scavenging activity, using methanol for broccoli extraction could also produce a satisfactory result. Therefore, it is concluded that methanol is the best solvent to extract antioxidants from broccoli. Besides, Eberhardt et al.^53^ stated that chemical estimates of antioxidant capacity within the plant may not accurately reflect the complex nature of the full antioxidant activity of broccoli extracts within cells, and these expectations were explained in our study: although the methanolic extract has the superiority in the concentration of most compounds, the biological antioxidant activity was nearly equal to the aqueous extract.

On the other hand, in the present study, methanolic broccoli extract showed antibacterial as well as anti-fungal activities against pathogens of human concern. In previous studies, it has been reported that broccoli crude extracts have activity against clinically significant bacteria^54,55^. Also, it was found that crude extract from raw and defrosted florets and stems has antimicrobial activity against bacteria and yeast that cause diseases in humans and plant pathogenic fungi since it showed an inhibitory effect against gram-positive (B. cereus, S. aureus, and S. xylosus) and Gram-negative (Proteus vulgaris, Shigella flexneri, and Shigella sonnei) bacteria and also against phytopathogenic fungi^56^. Furthermore, ethanolic broccoli extract showed antibacterial activity against some human pathogens, where Bacillus subtilis was found to be a more susceptible organism, followed by E. coli, S. pyogenes, S. aureus, P. aeruginosa, and K. pneumonia^57^. The in vitro antimicrobial activity of the methanolic broccoli extract can be attributed to the abundance of phytochemical compounds that are known for their potent action against a wide variety of microorganisms, like Naringenin^58^, Quercetin^59^ and Resveratrol^60^.

DMBA, as an indirect environmental contaminant, can induce oxidative stress^61^. Firstly, it binds to the aryl hydrocarbon receptor (AhR) in the cytosol^62^. Then it binds to xenobiotic response elements located in the promoter region of the cytochrome P450 (CYP 450) gene, inducing its expression^63^. Cytochrome p450 metabolized DMBA to more aggressive diol-epoxides that attack different macromolecules such as protein, lipid, and DNA^2^, increasing lipid peroxidation with a decrease in antioxidant defense mechanisms and tissue damage^12^. The progression of oxidative stress may cause various types of RNA and DNA damage like strand breaks and base lesions^64^.

In vivo*,* DMBA is transformed into DMBA-3,4-diol-1,2-epoxide (DMBA-DE) in the liver and breast, altering tissue redox equilibrium and causing oxidative stress. This mechanism starts with the binding of DMBA to the aryl hydrocarbon receptor (AhR) in the cytosol^62^. Then it binds to xenobiotic response elements located in the promoter region of the cytochrome P450 (CYP 450) gene, inducing its expression^63^. Cytochrome p450 metabolized DMBA to more aggressive diol-epoxides that attack different macromolecules such as protein, lipid, and DNA^2^, increasing lipid peroxidation with a decrease in antioxidant defense mechanisms and tissue damage^12^.

Reactive oxygen species (ROS) generated cause damage to the DNA or protein cell cycle, which can cause unregulated cell division and growth and cancer initiation^64,65^. Therefore, the detrimental impacts of DMBA in different tissues and the protective role of the broccoli extracts were assessed in the current investigation. GSH is one of the oxidative stress biomarkers that was decreased in the liver, kidney, and brain of DMBA-treated rats compared to the control. DMBA decreased GSH concentrations in the brain (Mutlu and Baltaci,^66^), the liver^67,68^, and the kidney Dakrory et al.^69^. It is worth noting that GSH is a non-protein thiol that has a role in scavenging the electrophilic moieties involved in the initiation and pathogenesis of different diseases^70–72^.

Lipid peroxidation (LPO) is one of the most important expressions of oxidative stress induced by ROS. Malondialdehyde (MDA) is an indicator of LPO that increases in various diseases and is reported to be associated with DMBA-induced toxicity^73,74^. The current study's findings that DMBA intoxication boosted hepatic, renal, and brain MDA are consistent with those of Paliwal et al.^75^ for the liver, Dakrory et al.^69^ for the kidney, and Mutlu and Baltaci,^66^ for the cerebral cortex. DMBA toxicity comes from its metabolism, which produces reactive metabolites that can produce free radicals with an elevation of oxidants than antioxidant capacity, generating oxidative stress^76^.

Several studies stated that DMBA increased ROS production, provoking the pathogenesis of apoptosis^77^. The apoptosis-like mechanism of DMBA is confirmed in the current study by DNA fragmentation, which was increased in different tissues of rats treated with DMBA, and these results were compatible with those of Prasad et al.^78^ regarding the mouse adrenal cortex. Meanwhile, in the present study, broccoli extracts co-supplementation mitigated oxidative stress by enhancing GSH concentration and diminishing MDA and DNA fragmentation.

These results are in agreement with different studies that confirmed the antioxidant activities of broccoli extracts through decreasing oxidative stress by increasing GSH on critical antioxidants for scavenging lipid peroxides in diabetic rats^79,80^ and type 2 diabetic patients^81^. Also, Wu and Juurlink^82^ and Wu et al.^83^ showed an elevation in glutathione concentration after the administration of dried broccoli sprouts in rats. Moreover, Bahadoran et al.^81^ demonstrated that sulphoraphane exposure has a protective effect on the colon cell line against benzo (a) pyrene DNA strand breaks. In general, brassica extracts and their components promote antioxidant and DNA protection effects against different carcinogens^84^. Both the aqueous and methanolic extracts showed nearly the same chemoprotective effect against the oxidative stress induced by DMBA and their potency to restore the antioxidant status In vivo. Broccoli is rich in substances that have great antioxidant effects, like kaempferol^85^, resveratrol^86^, caffeic acid^87,88^, naringenin^89^, chlorogenic acids^90^, caffeic acid^91^, syringic acid^92^, ferulic acid (Zduńska et al.^93^).

The effect of DMBA on the expression level of several genes associated with the anti-apoptotic gene (bcl2) and proto-oncogenic gene (c-Fos) was investigated in the present study. BCL2 are anti-apoptotic proteins that, together with pro-apoptotic proteins (Bax, Bak, Bad, and Bim), control the intrinsic apoptotic pathway^94^. In the current study, the expression level of bcl2 in DMBA-treated rats was not affected in the liver (Fig. 4a), but was upregulated in the kidney and downregulated in the brain. In the same vein, Abdel-Rahman et al.^95^ reported a significant increase in renal Bcl-2 in DMBA-treated rats. The elevation of BCL2 expression in the kidney may indicate that DMBA suppresses apoptosis after the NF-B activation signaling pathway is followed by cytochrome c release blocking^96^. The decrease in the expression level of bcl2 in the brain is supported by the literature^51,97^. The lipophilic nature of DMBA in the brain induces apoptosis that is provoked by oxidative stress, leading to loss of mitochondrial integrity, cytochrome c release, and capacity activation, triggering progressive degeneration and death of neuronal cells^98^.

To our knowledge, our study is considered the first concern regarding the effect of DMBA on the expression of c-Fos in different organs, where the level of c-Fos expression was non-significant in the rat liver, kidney, and brain. In response to external stimuli, the gene c-Fos plays a role in cell proliferation and differentiation; nevertheless, dysregulation of this gene has been linked to the development of cancer. Recently, it was revealed that c-Fos serves a significant function in neurogenesis, and in comparison to c-Fos + / + mice, adult c-Fos −/− mice have a 40–60% loss in body weight and a noticeably smaller brain. (Velazquez et al.^99^).

In the present study, the co-supplementation of broccoli extracts affected the expression of BCL2 and c-Fos genes. In the current study, the expression of BCL2 significantly decreased in co-supplementation with aqueous broccoli extract in all organs, while its expression in methanolic broccoli extract significantly increased in liver and brain tissues.

These results agreed with the observation of Priya et al.^100^, who found a significant decrease in BCL2 accompanied by an increase in Bax and Caspase-3 levels after oral administration of isothiocyanate-SFN against B(a)P in Swiss albino mice. This observation is in line with Wang et al.^101^, who showed the ability of sulforaphane to downregulate the expression of Bcl-2, which suppresses apoptosis, and showed activation of caspases to complete apoptosis in prostate cancer cells. So, the upregulation of Bax, downregulation of Bcl-2, and activation of caspases 3, 9, and 8 were involved in sulforaphane-induced cell apoptosis. On the other hand, Mukherjee et al.^102^ demonstrated that broccoli helps in the reduction of pro-apoptotic Bax protein and the induction of the expression of anti-apoptotic Bcl2 and Bcl-xL proteins in ischemic Sprague–Dawley (SD) male rats administered steamed and cooked broccoli. These results suggest that the methanolic extract of broccoli in our current study promotes the survival antioxidant pathway, while the aqueous extract of broccoli enhances apoptosis and death-driven proteolytic proteins against any DMBA-damaged cells.

In the current study, the expression of c-Fos significantly increased with co-supplementation of both extracts with DMBA. This result could be explained by Shen et al.^103^, who demonstrated that sulforaphane activates ERK1/2. The ERK signaling pathway is a member of the mitogen-activated protein kinase (MAPK) family that, when activated by phosphorylation of ERK1/2 through sulforaphane lead to an increase in c-Fos transcription factors^104,105^. In this context, the sulforaphane biologically active component of broccoli, helps in the activation of the ERK/MAPK signaling pathway that is involved in the induction of transcription factors such as c-Fos^103,105^.

From a histopathology and immunohistochemistry point of view in the present study, these assessments have confirmed all the previous results that showed that administration of DMBA to rats resulted in severe pathological alteration in the liver, similar to previous studies that showed severe liver damage due to DMBA^106^.

Since DMBA is mainly metabolized in the liver by P450 enzymes, it forms toxic metabolites that cause the formation of DNA adducts. Therefore, the liver was the most affected organ and showed the highest NF-κβ P65 expression. NF- is a crucial transcriptional regulator of inflammation and regulates the inflammatory signaling pathways in the liver^107,108^. NF-B signaling and crosstalk play a role in several steps of carcinogenesis, such as the establishment of its remodeling to the precancerous niche (PCN) and the transition of a normal cell to a cancer cell (Brücher et al.^109^). NF-B activity was increased in association with cancer development in transgenic mice^110^. NF-B constitutive activation was found to attenuate p53 and promote carcinogenesis. NF-B activity was also recorded in several cancers (Brücher et al.^109^). Remarkably, the expression of NF-B was decreased by the administration of aqueous broccoli and methanolic extract, which indicates that they have a hepatoprotective role against the toxic and carcinogenic effects of DMBA by targeting the NF-B pathway. DMBA also resulted in mild renal histopathological alteration, similar to previous studies^111^. Moreover, NF-B expression was observed in mesangial cells of the glomeruli in the present study. Different pathophysiological conditions trigger NF-B in renal cells, in which its expression is associated with experimental and human kidney diseases, and its activation can be modulated in vivo and in vitro by pharmacological maneuvers^112^. Aqueous broccoli and methanolic extract administration in the present study managed to decrease NF-B expression in the kidney and ameliorate the histopathological alteration. The brain also showed histopathological lesions due to DMBA administration. It was reported before that DMBA and its metabolites can cross the blood–brain barrier and induce the expression of CYP P450 in endothelial cells of blood–brain interfaces^113^ thus resulting in neurotoxicity. NF-B is a crucial regulator of neuronal morphology^114^. In DMBA-intoxicated rats in the current study, NF-κB was observed in glial cells. NF-B was reported to be inducible in glial cells and regulate inflammatory processes that exacerbate diseases such as autoimmune encephalomyelitis, ischemia, and Alzheimer's disease; therefore, its inhibition in glia could ameliorate disease. The use of aqueous broccoli and methanolic extract was able to counteract the toxic effect of DMBA on the brain, in which the histopathology was improved and NF-B expression was decreased in glial cells. Among the polyphenols that have anti-inflammatory effects via modulating NF-B are resveratrol (Xu et al^115^) and quercetin (Chekalina et al.^116^). In the current study, γ-catenin expression was increased in the liver and kidneys of DMBA-treated rats. Plakoglobulins *(γ-catenin* and β*-catenin*) function in cell adhesion and the Wnt signaling pathway. It was implied, based on available evidence, that both β—and γ*-catenin* may play distinct roles in cancer through differential effects on downstream target genes. Both β- and γ-catenin expression was increased in the cytoplasm of carcinomas^117^. Similarly, another study showed that cell adhesion molecules such as α-catenin, β-catenin, γ-catenin, E-cadherin, and integrin were expressed at a higher protein level in the Alpha5 and Tumor2 cell lines compared to non-tumorigenic cell lines^118^. Therefore, the use of the carcinogenic DMBA resulted in increased protein expression of γ-catenin, whereas it was decreased in the groups protected with aqueous and methanolic extracts of broccoli. The anticarcinogenic effect of broccoli is attributed to the presence of bioactive compounds that modulate the Wnt/β-catenin pathway.Resveratrol was reported to affect cell migration through the PI3K/Akt and Wnt/catenin signaling pathways (Tsai et al^119^).

## Conclusion

Broccoli as a dietary component showed a strong green chemoprotective effect against oxidative stress, DNA damage, and genotoxicity induced by DMBA intoxication in rats by increasing the concentration of GSH and decreasing MDA concentration, and DNA fragmentation percentage, and BCL2 gene expression with amileoration histopathological changes and downregulating immunoexpression of NF-κβ P65 and γ-catenin. In addition to the in vitro antimicrobial activity against different bacteria and fungi of clinical importance, this could help in further applications to overcome the excessive use of antibiotics and the development of multi-drug-resistant strains (MRES). Also, it’s worth noticing that methanolic broccoli extract has superior effects to the aqueous one*.* However, additional investigations are warranted to further explore the biochemical and molecular pathways underlying broccoli's protective effects.

### Supplementary Information

Supplementary Figure S1.

## Data Availability

All data generated or analysed during this study are included in this published article**.**

## Abbreviations

AhR: Aryl hydrocarbon receptor
AMB: Amphotericin B
AQ group: Aqueous group
AQ-DMBA group: Aqueous DMBA group
B(a)p: Benzo(a)pyrene
BAX: Bcl-2-associated X protein
BCL2: B-cell lymphoma 2
BCL-XL: B-cell lymphoma-extra large
c-fos: Proto-oncogene
CN: Gentamicin
CT: Colistin sulphate
CYP: Cytochromes P450
DMBA: 7,12 Dimethyl Benz (a) anthracene
DPPH: 2,2′-Diphenyl-1-picrylhydrazyl radical
GSH: Reduced glutathione
GST: Glutathione- s- transferase
HPLC: High performance liquid chromatography
MDA: Malondialdehyde
Meth group: Methanolic group
Meth_DMBA group: Methanolic DMBA group
MRSA: Methicillin-resistant *Staphylococcus aureus*
NF- κβ: Nuclear factor κβ P65
PAHs: Polyaromatic hydrocarbons
ORAC: The oxygen radical absorbance capacity
PCN: Precancerous niche
ROS: Reactive oxygen species
MAPK: Mitogen-activated protein kinase
ERK: Extracellular signal-regulated kinase
RT: Retention time
B(a)p: Benzo(a)pyrene
SD rats: Spargue Dawley rats
SFN: Sulforaphane
VA: Vancomyci
