# Supplementary material for: Potential chemopreventive effects of Broccoli extract supplementation against 7, 12 dimethyl Benz(a)anthracene (DMBA) -induced toxicity in female rats
Source: Sci Rep. 2023 Oct 11;13:17234. doi: 10.1038/s41598-023-43629-2 (PMC10567736; doi:10.1038/s41598-023-43629-2)
Supplement: Supplementary file 1 — Supplementary Figure S1. [file 41598_2023_43629_MOESM1_ESM.docx]

**Supplementary Materials:**

**DNA fragmentation % in different groups.**

**a)**

**b)**

**c)**

**d)**

**Supplementary Fig. S1.** Electrophoretic mobility of different organs fragmented DNA in different groups on 1% **uncropped** agarose gel.a)Liver organ.b)Kidney organ.c) brain organ. d) DMBA groups in in liver ,kidney , and brain organs
